# Altered cerebrospinal fluid-based clearance mechanisms in aging autistic adults

**DOI:** 10.21203/rs.3.rs-9450979/v1

**Published:** 2026-04-20

**Authors:** Danielle Christensen, Giuseppe Barisano, Bradley J. Wilkes, Young Seon Shin, Jingying Wang, Ellen Parks, Ann-Marie Orlando, Bikram Karmakar, Stephen A. Coombes, Stefano Sotgiu, Zheng Wang

**Affiliations:** University of Florida; Stanford University; University of Florida; University of Florida; University of Florida; University of Florida; University of Florida; University of Wisconsin–Madison; University of Florida; University of Sassari; University of Florida

## Abstract

**Background.:**

Autistic adults demonstrate a 4–6-fold increased risk of unspecified dementia compared with the general population; however, the neurobiological substrates underlying this elevated risk remain unexplored. Alterations in cerebrospinal fluid–based mechanisms involved in brain metabolic waste clearance may represent a shared neuropathological pathway between autism spectrum disorder and dementia. Specifically, developmental deviations in cerebrospinal fluid-related imaging markers have been consistently reported in autistic infants, children, and adolescents, and brain amyloid and other metabolic waste accumulation is a hallmark of Alzheimer’s disease and related dementias. Despite this overlap, cerebrospinal fluid-based regulatory mechanisms have not been systematically examined in ageing autistic adults. Here, we used a multimodal magnetic resonance imaging approach to quantify structural and diffusion-based markers of cerebrospinal fluid regulation in middle-aged and older autistic adults compared with matched controls.

**Methods.:**

Forty-nine autistic adults aged 30–73 years and 61 age-, sex-, and intelligence quotient–matched controls underwent T1-, T2-, and diffusion-weighted imaging. Measures included white matter perivascular space volume fraction, count fraction, and mean diameter; diffusion-based indices of fluid movement along perivascular pathways; and volumes of the lateral ventricles and choroid plexus.

**Results.:**

With increasing age, autistic adults exhibited significantly greater increases in white matter perivascular volume fraction within the left inferior parietal lobule compared with controls. Autistic adults also showed significantly reduced diffusion indices and larger bilateral lateral ventricle and choroid plexus volumes relative to controls. Across both groups, increasing age was associated with higher white matter perivascular volume fraction in the right pars triangularis, reduced diffusion indices, and enlargement of the bilateral lateral ventricles and left choroid plexus.

**Limitations.:**

First, the cross-sectional design limited our ability to quantify intra-individual variability and capture longitudinal trajectories. Second, the sample primarily comprised cognitively unimpaired autistic adults. Third, participants were predominantly of average or above-average intelligence; thus, findings may not generalize to autistic adults with ID. Finally, health factors including sleep disturbance, cardiovascular and metabolic disease, polypharmacy, and lifelong medication exposure, may have influenced these findings. Future large-scale studies should systematically evaluate their potential confounding and moderating effects.

**Conclusions.:**

These findings demonstrate that ageing autistic adults exhibit convergent alterations in cerebrospinal fluid regulatory mechanisms, reflected in perivascular space morphology, diffusion-based fluid dynamics, and ventricular and choroid plexus enlargement. Together, the results link early developmental deviations to later-life vulnerability and highlight cerebrospinal fluid dysregulation as a potential candidate neurobiological substrate contributing to the increased prevalence of dementia in autistic adults.

## Background

Autistic adults exhibit a 4–6-fold increased risk of unspecified dementia relative to the general population.^1–5^ This elevated risk persists after adjustment for intellectual disability (ID), psychiatric comorbidities, and lifestyle-related factors, suggesting that autism spectrum disorder (ASD) independently confers a heightened neurobiological vulnerability to pathological brain ageing. While the precise mechanisms underlying this heightened risk have not yet been identified, multiple dementia subtypes—including Alzheimer’s disease, vascular dementia, and frontotemporal dementia—exhibit broad alterations in CSF-based mechanisms critical for brain metabolic waste clearance.^6–9^ These alterations include enlarged white matter perivascular spaces (WM-PVS),^10–14^ reduced indices of fluid diffusion along perivascular spaces,^15–17^ and larger volumes of the lateral ventricles^18–20^ and choroid plexus^21–23^. Evidence of dysregulation in CSF-based mechanisms has been reported in middle-aged and older autistic adults, including increased extracellular free-water in frontal lobe transcallosal white matter^24^ and enlargement of the cerebral ventricles compared to age-matched neurotypical controls.^25^ However, these findings have been derived from single imaging markers rather than from a comprehensive multimodal approach to examine CSF-based mechanisms. Given that ageing neurobiology in ASD remains critically under-researched, the elevated prevalence of dementia in autistic adults underscores the need for systematic investigation of pathological brain ageing processes across middle and later life. Recent work has recognized the pathophysiological overlaps between ASD and Alzheimer’s disease, emphasizing shared alterations among perivascular pathways and brain waste clearance mechanisms.^26^ Together, these findings suggest that alterations in CSF-based mechanisms involved in brain metabolic waste clearance may represent an overlapping substrate linking ASD and dementia-related processes, offering insight into shared neurobiological pathways driving this increased prevalence.

Proper functioning of CSF-based mechanisms is necessary for the efficient removal of neurotoxic proteins that may otherwise accumulate within the parenchyma over time.^7,27,28^ Brain metabolic waste clearance relies on the bulk fluid exchange between CSF and interstitial fluid along the perivascular spaces (PVS).^8,27,29^ CSF is produced by the choroid plexus, a specialized structure lining the brain’s ventricular system,^30,31^ and is derived from arterial blood plasma.^31^ Beyond CSF production, the choroid plexus also regulates nutrient transport, metabolic exchange between blood and CSF, and triggering neuroimmune responses in the central nervous system.^31–34^ The lateral ventricles are the primary compartments that support continuous CSF circulation and maintain homeostasis.^35,36^ CSF circulates through the ventricular system and exits into the subarachnoid space as extra-axial CSF (EA-CSF).^37^ From here, EA-CSF flows along perivascular pathways that surround penetrating brain arterioles^38,39^ and contributes to CSF-interstitial fluid exchange,^27,40,41^ facilitating metabolic waste removal throughout the parenchyma.^7,27,28^ Structurally, PVS are the fluid-filled compartments bounded internally by the abluminal surface of arterioles and externally by a sheath of astrocytic endfeet,^27,42,43^ which are specialized glial processes extending from astrocytes.^27^ Cardiac-driven arterial pulsatility offers the mechanical force that propels CSF movement along PVS.^40,44^ This rhythmic contraction and relaxation of the arteriole wall promotes CSF-interstitial fluid exchange and enables metabolic waste removal from the surrounding tissue,^27,44^ directing collected byproducts toward perivenous drainage pathways.^45^ This fluid-based mechanism, commonly referred to as the glymphatic system, is estimated to remove over half of extracellular amyloid-β from the brain, underscoring its critical role in minimizing the accumulation of neurodegeneration-promoting products.^8,46^

Developmental alterations in CSF-based mechanisms are well-documented in ASD,^47–56^ yet remain largely unexplored in autistic adults. In infants at high risk for ASD, elevated extra-axial CSF^49^ and widespread PVS enlargement^52^ have been shown to predict later ASD diagnosis. In autistic children aged 1–9 years, increased WM-PVS count and volume fraction have been associated with greater clinical severity of ASD.^47^ Diffusion tensor imaging analysis along the perivascular space (DTI-ALPS) of deep medullary veins has further revealed lower indices in autistic children aged 2–5 years compared with neurotypical controls, suggesting reduced fluid transport along these pathways.^53^ Converging structural MRI studies have also demonstrated enlarged lateral ventricles and choroid plexus in autistic children and adolescents relative to age-matched peers,^54,55,57^ suggesting that altered CSF-based mechanisms in ASD are reflected not only in downstream PVS morphology and fluid transport, but also in upstream CSF-handling. Thus, enlargement of the lateral ventricles and choroid plexus implicates abnormalities in structures involved in both CSF handling as well as solute filtration and immune signaling between blood and CSF.^31–34^ Despite ample evidence of early-life alterations, little is known about how CSF-based features manifest in ASD after childhood, particularly in middle-aged and older autistic adults. Given the critical role of CSF regulation in brain metabolic waste clearance,^7,27^ this knowledge gap limits the development of a mechanistic framework for understanding the heightened vulnerability to dementia in this understudied clinical population.^47^

In this multimodal imaging study, we used structural and diffusion-based MRI approaches to compare WM-PVS count (the total number of PVS lesions), volume (total lesion volume), and diameter (mean diameter of detected PVS) across the frontal, parietal, temporal, and occipital lobes, as well as DTI-ALPS indices and volumes of the lateral ventricles and choroid plexus, between middle-aged and older autistic adults and matched neurotypical controls. To our knowledge, no prior study has jointly examined WM-PVS morphology, DTI-ALPS, and volumes of the lateral ventricles and choroid plexus in ageing autistic adults. Consistent with findings in autistic infants and children,^47,53–55^ we predicted that CSF dysregulation would persist beyond early development and hypothesized that autistic adults would exhibit 1) greater WM-PVS volume, count, and diameter; 2) lower DTI-ALPS indices; and 3) increased lateral ventricle and choroid plexus volumes relative to controls. We further hypothesized that age-associated changes in these measures would be more pronounced in autistic adults compared with neurotypical controls, reflecting the life-course neurobiological vulnerability associated with ASD.

## Materials

### Participants

A total of 49 autistic adults and 61 neurotypical controls (NT) participated in this cross-sectional study. The two groups were matched on age, sex, and full-scale IQ (FSIQ), performance IQ (pIQ), and verbal IQ (vIQ) (Table 1). Autistic participants were recruited through the Center for Autism and Related Disabilities (CARD) at the University of Florida, the University of Central Florida, and the University of South Florida, as well as the SPARK Research Match program (https://www.sfari.org/resource/research-match). Controls were primarily recruited through local flyers and word of mouth referrals. All study procedures were approved by the Institutional Review Boards (IRB) at the University of Florida (IRB202100659, approved September 23, 2021). In accordance with the Declaration of Helsinki, written informed consent was obtained from all participants after they received a complete description of the study and its procedures.

Screening criteria for participants were consistent with those used in previous studies from our lab and others ^58,59^. Autistic adults with a clinical diagnosis of ASD were required to score > 32 on the Autism Spectrum Quotient for Adults (AQ-50)^60^ and ≥ 65 on the Social Responsiveness Scale Adult Self-Report (SRS-2)^61^ to be invited for a comprehensive in-person diagnostic evaluation using the Autism Diagnostic Observation Schedule, Second Edition (ADOS-2) ^62^. Diagnosis of ASD was confirmed through an integrative review of AQ-50, SRS-2, and ADOS-2 scores, in conjunction with expert clinical judgement by a licensed clinician, following the DSM-5 criteria.^63^

Neurotypical controls were recruited if they scored ≤ 22 on the AQ-50 and < 60 on the SRS-2. Exclusion criteria for controls included a family history of ASD or related neurodevelopmental conditions among first- and second-degree relatives.

Exclusion criteria for both groups included: (1) a diagnosis of intellectual disability (including non-specific developmental delay), mild cognitive impairment, or dementia; (2) a current or past major psychiatric condition (e.g., schizophrenia, bipolar disorder, or post-traumatic stress disorder); (3) a current or past illness involving the central nervous system (e.g., brain tumor, thyroid disease, Cushing’s disease, or HIV infection); (4) a diagnosed neurological disorder (e.g., Parkinson’s disease, cerebellar ataxia, seizure, dystonia, or stroke); (5) a family history of heritable neurological disease (e.g., Huntington’s disease, Wilson’s disease, or amyotrophic lateral sclerosis); (6) implanted medical devices incompatible with MRI (e.g., cardiac pacemakers, infusion pumps, cochlear implants); (7) current pregnancy; (8) FSIQ < 75 as measured by the Wechsler Abbreviated Scales of Intelligence, Second Edition ^64^, or (9) non-English-speaking status.

Medication use and polypharmacy are prevalent in ASD ^65^. To allow an ecologically representative sample of autistic adults, routine medication use was not an exclusion criterion in this study. Participants taking psychotropic medication included those prescribed antidepressants (ASD = 25, NT = 4), antipsychotics/neuroleptics (ASD = 4), sedatives/hypnotics/anxiolytics (ASD = 7, NT = 2), stimulants (ASD = 9), and anticonvulsants (ASD = 6, NT = 1).

### MRI data acquisition

MRI scans were acquired using a 3T Siemens Prisma scanner equipped with a 64-channel head coil at the University of Florida McKnight Brain Institute. Participants were screened before scanning, and hearing protection was provided prior to entering the scanner room. Foam padding was securely placed around the head to minimize motion during image acquisition. For T1-weighted imaging, the MPRAGE sequence was acquired with the following parameters: repetition time (TR) = 2000 ms, echo time (TE) = 2.99 ms, flip angle = 8°, field of view (FOV) = 256 × 256 mm, matrix size = 320 × 320, 208 sagittal slices, and isotropic voxel size = 0.8 × 0.8 × 0.8 mm^3^. For T2-weighted imaging, a three-dimensional SPACE sequence was collected with the following parameters: TR = 2500 ms, TE = 370 ms, variable refocusing flip angle, FOV = 256 × 256 mm, matrix size = 320 × 320, 208 sagittal slices, and isotropic voxel size = 0.8 × 0.8 × 0.8 mm^3^. Diffusion weighted scans were acquired with an echo-planar imaging sequence with the following parameters: TR = 6400 ms, TE = 58 ms, b-values: 5 × 0 s/mm^2^ and 64 × 1000 s/mm^2^, bandwidth = 2442 Hz/pixel, FOV = 256 × 256, matrix size = 128 × 128, 69 axial slices, and isotropic voxel size = 2.0 × 2.0 × 2.0 mm^3^.

### White matter perivascular spaces

**Figure 1:**
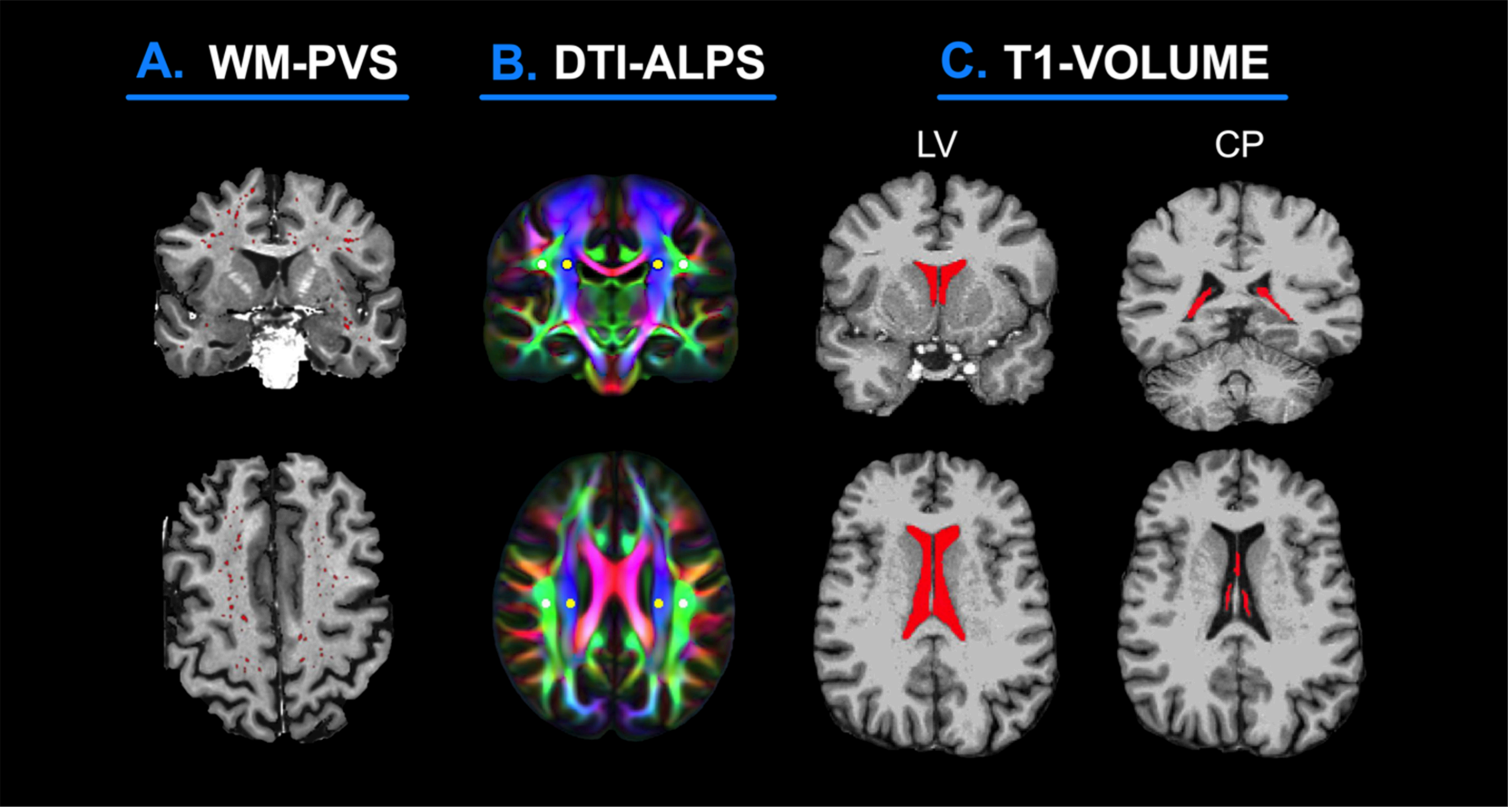
Coronal and axial MRI depictions of multimodal methods used in this study to examine CSF-based mechanisms. **(A)**Automated segmentation of white matter perivascular spaces (WM-PVS). **(B)**Diffusion tensor imaging analysis along the perivascular space (DTI-ALPS). **(C)**Volumetric segmentation of the lateral ventricles (LV) and choroid plexus (CP).

T2-weighted images were visually assessed by the team’s physician-scientist (GB) for PVS segmentation. Cases with prominent imaging artifacts that significantly compromised accurate PVS quantification were excluded from further analysis (ASD = 2). T2-weighted images were rigidly registered to the processed T1-weighted images using a six-degree-of-freedom transformation and boundary-based cost function, as implemented by the *bbregister* command using FreeSurfer version 8.0.0. We segmented MRI-visible WM-PVS using a robust, fully automated pipeline that has been previously validated across multiple independent datasets of healthy aging and neurodegeneration ^10^ (Fig. 1A). Briefly, we first generated enhanced perivascular contrast (EPC) images for each participant by computing the voxel-wise ratio of T1-weighted to T2-weighted images ^66^. Prior work has demonstrated that this approach enhances PVS contrast and increases the sensitivity of both visual detection and automated PVS segmentation ^66^. We then applied a multiscale *Frangi vesselness* filter ^67^ to the white matter mask applied on the EPC images to enhance tubular structures consistent with PVS morphology. We set the filter parameters to α = 0.5 and β = 0.5, with c defined as half the maximum Hessian norm, in accordance with established recommendations ^66^. The *Frangi* filter assigns a *vesselness* value to each voxel based on the eigenvalues of the Hessian matrix, thereby preferentially improving elongated, vessel-like structures while suppressing non-tubular signal.

We applied the *Frangi* filter within 32 bilateral white matter regions generated using FreeSurfer according to the Desikan-Killiany atlas. To minimize contamination from non-PVS pathology, we first segmented white matter lesions unrelated to PVS using a fully automated approach ^68^ and excluded these lesions from the white matter masks prior to *vesselness* filtering, consistent with prior work ^10^. We then automatically derived PVS masks from the resulting *vesselness* maps using a percentile-based thresholding approach that has been previously validated ^69^.

For each participant, we quantified PVS metrics including total count, total volume, and mean diameter. Total count and total volume refer to the occurrence and spatial occupancy that visible PVS exhibit in a mask. Mean diameter refers to the average cross-sectional width of detected PVS ^69^. We derived these measures using MATLAB’s *regionprops3* function applied to the binarized PVS masks. Because both PVS count and volume scale with the volume of the underlying white matter region, we normalized these measures by the corresponding regional white matter mask volume to derive PVS count fraction and PVS volume fraction, respectively^69^.

We grouped white matter masks from the left and right hemisphere by anatomical lobe, consistent with prior studies in autistic children ^70^. The frontal lobe comprised 11 masks, including the superior frontal gyrus, rostral middle frontal gyrus, caudal middle frontal gyrus, pars opercularis, pars orbitalis, pars triangularis, lateral orbitofrontal gyrus, medial orbitofrontal gyrus, precentral gyrus, paracentral lobule, and frontal pole. The parietal lobe consisted of 5 masks encompassing the postcentral gyrus, supramarginal gyrus, superior parietal lobule, inferior parietal lobule, and precuneus regions. The temporal lobe included 8 masks incorporating the entorhinal cortex, parahippocampal gyrus, fusiform gyrus, superior temporal gyrus, middle temporal gyrus, inferior temporal gyrus, transverse temporal gyrus, and temporal pole. Finally, the occipital lobe consisted of 4 masks encompassing the lingual gyrus, pericalcarine cortex, cuneus, and lateral occipital cortex. Across all bilateral regions of interest (ROIs) and PVS metrics, each participant yielded 168 PVS variables (56 ROIs × 3 PVS metrics).

### DTI-ALPS index

We calculated the DTI-ALPS index using an MNI-space approach ^15^ (Fig. 1B) and processed diffusion weighted MRI data following pipelines used in previous published work ^24^. Specifically, we performed denoising and Gibbs artefact removal using MRtrix3 ^71^. We then generated brain masks directly from the diffusion weighted images using the *dwi2mask* function in MRtrix3 ^71^. Next, we corrected for eddy current induced image distortions and head motion, with corresponding adjustment to gradient directions (i.e. b-vectors) using Eddy, a fully automated quality control framework in FSL ^72^.

We subsequently reconstructed diffusion tensors using FSL’s *dtifit* function to generate fractional anisotropy (FA) images in native subject space. We registered the resulting FA images to the HCP1065 FA template ^73^ using the symmetric normalization (SyN) algorithm implemented in Advanced Normalization Tools (ANTs) ^74^, which applies both an affine transformation and a nonlinear diffeomorphic warp. These registered FA images in MNI space were visually inspected to confirm appropriate data quality, as in our prior work ^24^. We then applied the resulting transformation matrices to each of the six unique tensor elements (Dxx, Dxy, Dxz, Dyy, Dyz, and Dzz) to transform the diffusion tensor into MNI space.

To compute the index, we defined four bilateral spherical regions of interest (ROIs; 5mm-diameter) at the level of the lateral ventricles to sample projection and association white matter fibers. We centered projection fiber ROIs at MNI coordinates (± 25, − 20, 28) and association fiber ROIs at (± 39, − 20, 28), based on the HCP1065 1mm FA template and corresponding tensor maps overlaid in RGB to confirm proper orientation. We then calculated the DTI-ALPS index as the ratio of diffusivity along the x-axis to diffusivity perpendicular to the primary fiber direction, defined as:

$$DTI-ALPS=\frac{\mathrm{mean}(\mathrm{Dxx}\_\mathrm{proj},\;\mathrm{Dxx}\_\mathrm{assoc})}{\mathrm{mean}\left(\mathrm{Dxx}\_\mathrm{proj},\;\mathrm{Dxx}\_\mathrm{assoc}\right)}$$

where Dxx represents diffusivity along projection and association fibers, Dyy represents perpendicular diffusivity in projection fibers, and Dzz represents perpendicular diffusivity in association fibers. We averaged left and right hemisphere values to obtain a single bilateral ALPS index for each participant. Due to the timing in which individuals were recruited in this study, five participants in our sample were unable to receive the diffusion MRI necessary for DTI-ALPS analysis (ASD = 4; NT = 1).

### Lateral ventricle and choroid plexus volume

We performed quantification of lateral ventricle and choroid plexus volume using FreeSurfer versions 7.2.0 ^75^ and FSL version 6.0 ^72^ on a Linux-based computing system. First, T1-weighted images underwent slice-by-slice visual inspection across axial, coronal, and sagittal planes by multiple trained raters. Images that exhibited significant segmentation inaccuracies were corrected using the *Recon Edit* tool in FreeSurfer and subsequently cross-validated by an independent rater to ensure consistency. T1-weighted images that did not meet quality control criteria were excluded from further analysis (ASD = 1). Images that passed quality control were processed for volumetric segmentation and cortical surface reconstruction following the standard *recon-all* pipeline, which includes motion correction, intensity normalization, transformation to Talairach space, skull stripping and brain extraction, white matter segmentation, and cortical surface parcellation. We derived volumes of the left and right lateral ventricles and choroid plexus (Fig. 1C) from T1-weighted MRI scans using automated segmentation outputs generated by FreeSurfer. To account for inter-individual differences in brain size, we normalized these ROIs by the estimated total intracranial volume (eTIV) provided by FreeSurfer.

Participant sample size for each MRI measure was 49 ASD/61 NT for WM-PVS and T1-weighted volume, and 46 ASD/59 NT for DTI-ALPS.

### Statistical analysis

Statistical analysis was conducted in RStudio version 4.4.1. Demographic characteristics between autistic participants and neurotypical controls were compared using independent-sample *t*-tests for continuous variables, including age, FSIQ, pIQ, vIQ, and eTIV. Group differences in sex were assessed using a Chi-square test.

Normality of outcome variables was assessed using the Shapiro-Wilk test, which indicated non-normal distributions for several measures, including lateral ventricle and choroid plexus volumes, and approximately 40% of PVS variables. Given the non-normal distribution properties, linear regression models with 5000 permutations were implemented to examine main effects of group (ASD vs. NT), age, and their interaction (group × age). Each MRI metric served as the dependent variable in separate models. Age was mean-centered to improve interpretability of group and group-by-age effects and reduce multicollinearity among predictors.

Bonferroni correction was applied to adjust for multiple comparisons ^76^. For WM-PVS, Bonferroni thresholds were set within lobes (frontal, parietal, temporal, and occipital) to control the family-wise error rate while preserving anatomical specificity. Specifically, significance was defined as *p* < 0.00227 for the frontal lobe (22 masks), *p* < 0.00500 for the parietal lobe (10 masks), *p* < 0.00313 for the temporal lobe (16 masks), and *p* < 0.00625 for the occipital lobe (8 masks). Significance was defined as *p* < 0.025 for the lateral ventricles and choroid plexus volume, as each consisted of two ROIs (left and right), and *p* < 0.05 for the DTI-ALPS index.

## Results

### Participant demographics

Table 1 summarizes the demographic characteristics of the groups. Autistic adults and neurotypical controls were matched on age, sex, all IQ scores, and eTIV. Relative head motion also did not differ between groups.

### White matter perivascular spaces

**Figure 2:**
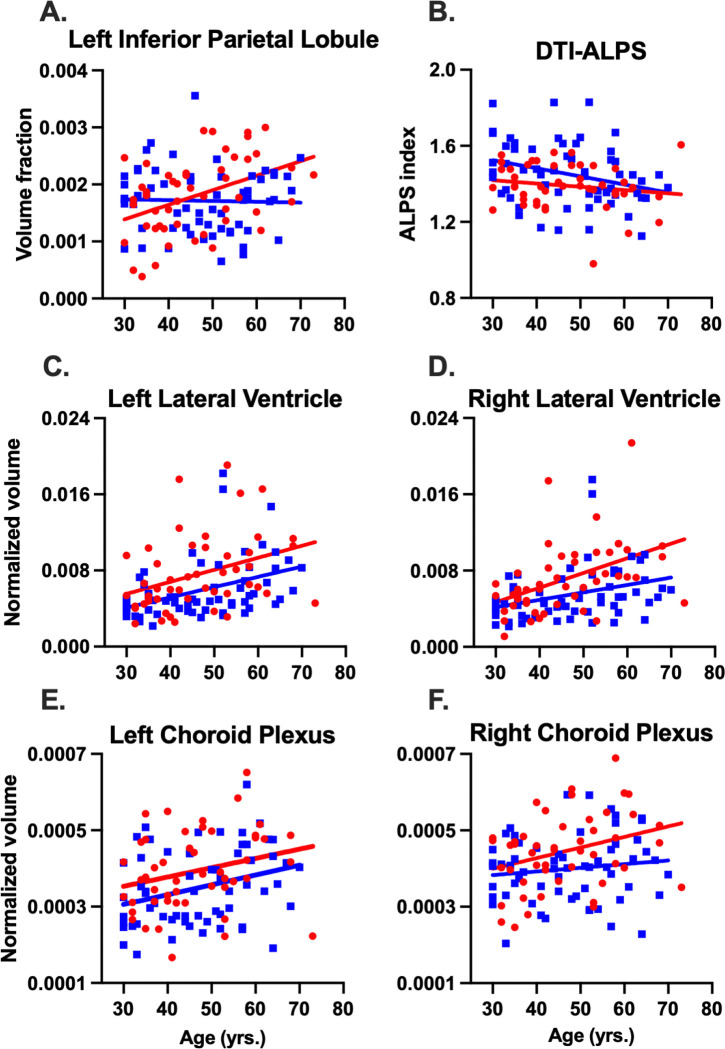
Scatterplots of significant group and group-by-age interactions across CSF-based mechanisms. Scatterplot of white matter perivascular space volume fraction in the left inferior parietal lobule with age **(A).** Autistic adults exhibited significantly steeper age-associated increases in WM-PVS volume fraction in the left inferior parietal lobule compared with neurotypical controls. Scatterplot of diffusion tensor imaging analysis along the perivascular space with age **(B).** Autistic adults exhibited significantly lower DTI-ALPS indices relative to neurotypical controls across middle and older adulthood. Scatterplots show normalized volumes of the left lateral ventricle **(C)**, right lateral ventricle **(D)**, left choroid plexus **(E)**, and right choroid plexus**(F)** with age. Autistic adults exhibited significantly larger left and right lateral ventricle and choroid plexus volumes compared to neurotypical controls.

Autistic adults exhibited significantly steeper age-associated increases in WM-PVS volume fraction within the left inferior parietal lobule compared to controls (Fig. 2). Furthermore, both groups demonstrated significant age-associated increases in WM-PVS volume fraction within the right pars triangularis (Table 2). Multiple lobar patterns in WM-PVS were demonstrated at the uncorrected level (*p-perm* < 0.05). For example, 1) group effects were most frequent in the temporal lobe (50%), followed by the parietal (25%) and occipital lobes (25%), with no group effect observed in the frontal lobe; 2) group × age interaction effects were most frequent in the parietal lobe (62.5%), followed by the temporal (25%) and frontal lobes (12.5%), and no interaction effects observed in the occipital lobe; and 3) age effects were predominantly found in the frontal lobe (55.6%), followed by the parietal (22.2%), temporal (18.5%), and occipital lobes (3.7%). Finally, hemispheric distribution showed that age and group main effects were bilaterally distributed, whereas all group × age interaction effects were left-lateralized. No other significant Bonferroni-corrected group, age, or group × age effects were observed (Supplementary Tables 1–4).

### DTI-ALPS index

Autistic adults showed significantly lower DTI-ALPS indices compared to controls (Fig. 2). Both groups showed significant age-associated decreases in DTI-ALPS indices, although no group × age interaction was observed (Table 3).

### Lateral ventricle and choroid plexus volumes

Autistic adults showed significantly larger lateral ventricle volumes in both the left and right hemispheres relative to controls (Fig. 2). Significant age-associated increases in left and right lateral ventricle volumes were observed in both groups; however, no significant group × age interactions were identified (Table 4). Autistic adults also demonstrated significantly larger left and right choroid plexus volume compared to controls, and both groups showed age-associated volume increases in the left choroid plexus. No significant group × age effect was observed in either the left or right choroid plexus.

## Discussion

Our study examined CSF-based mechanisms critical for brain metabolic waste clearance using multimodal imaging in ageing autistic adults and neurotypical controls. We report several novel findings. First, autistic adults exhibited an age-associated increase in WM-PVS volume fraction in the left inferior parietal lobule that was not observed in controls. Second, autistic adults demonstrated lower DTI-ALPS indices compared to controls. Third, autistic adults showed larger left and right lateral ventricles compared to controls. Fourth, autistic adults showed larger left and right choroid plexus volumes relative to controls. Fifth, both groups demonstrated greater WM-PVS volume fraction in the right pars triangularis, reduced DTI-ALPS indices, and increased bilateral lateral ventricle and left choroid plexus volumes with increasing age. Collectively, these findings provide evidence for a mechanistic overlap between CSF-based alterations observed in ageing autistic adults and those reported across multiple subtypes of dementia. Therefore, CSF-based alterations evident from early development through middle and older adulthood may support the framework of a lifelong neurobiological vulnerability contributing to increased dementia risk in ASD.

Compared to controls, autistic adults demonstrated an age-associated increase in WM-PVS volume fraction localized to the left inferior parietal lobule. The inferior parietal lobule is a densely vascularized region within the default mode network and serves as a major hub for sensory integration ^77^, language ^78^, and motor processes ^78^ through long-range connectivity across multiple cortical networks ^79^. Lateralized alterations of the left inferior parietal lobule have been reported in autistic children and adolescents in MRI studies of brain structure and functional connectivity, and have been strongly associated with clinical severity ^80,81^. Our finding extends this regional atypicality into middle and older adulthood, suggesting increased susceptibility of this integrative association cortex to perivascular burden with aging in ASD. As a metabolically demanding region, the inferior parietal lobule is particularly sensitive to vascular alterations that influence perivascular fluid dynamics, processes that are strongly modulated by arterial stiffness and pulsatility ^82^. Notably, the inferior parietal lobule is also recognized as an early site of vascular and metabolic dysfunction in Alzheimer’s disease, as evidenced by hypoperfusion, hypometabolism, and volume atrophy ^83–85^. Volumetric reductions of the left inferior parietal lobule have been shown to differentiate Alzheimer’s disease from normal cognition, with left-lateralized cortical thinning in this region identified as a prominent marker of early neurodegeneration ^85^. Although region-specific assessments of WM-PVS in Alzheimer’s disease remain limited, increased WM-PVS burden has been consistently documented ^13,69^. Taken together, these findings highlight a shared neurobiological substrate involving the left inferior parietal lobule and underscore its heightened vulnerability to pathological brain aging in autistic adults, warranting further investigation to clarify the mechanistic linkage between perivascular dysfunction and neurodegenerative risk in ASD.

Autistic adults exhibited lower DTI-ALPS indices relative to controls. The deep medullary veins, medullary arteries, and their associated perivascular spaces run predominantly in the x-direction (left–right orientation) at the level of the lateral ventricles. DTI-ALPS indices serve as a proxy measure of CSF-mediated fluid diffusivity along the perivascular pathways, supporting bulk CSF transport into and out of the brain parenchyma, capturing fluid movement toward both the brain surface and the ventricular system ^15,86^. Our findings indicate that reduced perivascular fluid diffusivity in ASD persists beyond childhood ^70^ and extends into middle and older adulthood. Lower DTI-ALPS indices have been reported across the dementia spectrum, including Alzheimer’s disease ^15,86^, vascular dementia ^87^, and Lewy body disease ^88^. In middle-aged adults, reduced DTI-ALPS indices predict future cognitive impairment and increased risk of dementia ^89,90^, highlighting the downstream consequences of chronically impaired fluid diffusivity. In Alzheimer’s disease, lower DTI-ALPS indices have been associated with increased cerebral amyloid-β deposition and worsened clinical outcomes, consistent with a disrupted capacity to regulate the accumulation of neurotoxic species ^91^. As a diffusion-based metric, DTI-ALPS indices may also reflect contributions from white matter microstructure alterations and vascular changes that are not specific to perivascular clearance ^15^. Nevertheless, reduced perivascular fluid diffusivity in ASD from early life ^50^ through older adulthood reflect enduring alterations in perivascular fluid regulation that may present lifelong altered clearance of neurotoxic byproducts along major medullary vessels within the brain parenchyma. This impairment aligns with converging evidence of central neuroimmune activation observed in the CSF ^92^ and brain tissue ^93^ of autistic children and adults. These findings emphasize functionally relevant alterations in perivascular fluid dynamics in aging autistic adults that may overlap with established biological vulnerabilities associated with dementia progression and coincide with observed differences in WM-PVS.

Autistic adults also demonstrated larger left and right lateral ventricle volumes relative to controls. This finding is consistent with prior studies reporting enlarged lateral ventricles in autistic children ^55^, adolescents ^54^, and adults ^58^, indicating that ventricular enlargement represents a persistent neurodevelopmental vulnerability in ASD across the lifespan. Expansion of the cerebral ventricles—particularly in cohorts in which ventriculomegaly is evident early in development—has been interpreted as reflecting compromised CSF homeostasis due to altered circulation, distribution, or resorption dynamics ^31^. This interpretation is particularly plausible in ASD, as ventricular enlargement has been observed very early in life, well before the typical onset of aging-related neurodegenerative processes. In parallel, ventricular enlargement has also been linked to broader structural alterations within surrounding brain regions ^58^ and has been observed in Alzheimer’s disease^18^, frontotemporal dementia ^94^, and Lewy body disease ^95^. Prior work from our group identified significant associations between enlarged ventricle volumes and reduced volumes in adjacent subcortical hippocampus and amygdala beginning in middle adulthood in ASD, but not during early development, highlighting accelerated structural deviations involving ventricular expansion and neighboring tissue volume reduction later in life ^58^. Notably, none of the autistic adults in the current study had a clinical diagnosis of mild cognitive impairment or dementia. Ventricular expansion observed in this cohort may reflect a persistent vulnerability in brain morphogenesis that may confer increased susceptibility to aging-related neurodegeneration rather than dementia-specific pathology in ASD ^54,55^. Future longitudinal studies are needed to disentangle the combined effects of early developmental vulnerability and aging-associated structural dynamics on ventricular enlargement in ASD.

Finally, autistic adults showed larger left and right choroid plexus compared to controls. This finding of choroid plexus enlargement is consistent with early childhood and adolescent studies of ASD ^55,56^, in which early brain overgrowth ^55^ and neuroimmune dysregulation ^56^ have been proposed as potential underlying mechanisms. Enlargement of the choroid plexus has also been commonly reported in aging-related neurodegenerative disorders, including Alzheimer’s disease^21^, vascular dementia ^96^, and frontotemporal dementia ^97^, implicating compromised CSF production, nutrient transport, metabolic exchange, neuroimmune signaling, and clearance of neurotoxic peptides between the blood and CSF compartments. Our finding in autistic adults suggests that choroid plexus enlargement is observable across middle and older adulthood, and may align with longstanding disruptions in CSF production, homeostasis and neuroimmune signaling contributing to increased vulnerability to aging-related neurodegeneration in autistic adults.

Age-associated patterns were observed in both groups across multiple neuroimaging markers. The age-associated increase in WM-PVS volume fraction in the right pars triangularis across both groups suggests that perivascular morphology within this region is particularly sensitive to aging independent of diagnosis. Furthermore, the presence of age-associated reductions in DTI-ALPS demonstrates that decreased fluid diffusivity along perivascular pathways represents a general feature of aging for both groups. The age-associated increases in left and right lateral ventricle volumes we observed in both groups is consistent with studies of neurobiological aging in the general population showing bilateral ventricular enlargement as a common feature of advancing age ^98^. Finally, both groups demonstrated a lateralized age-associated volume increase in the left choroid plexus, suggesting an asymmetric susceptibility to enlargement in this CSF-producing and immune-signaling region with increasing age.^31^

## Limitations

Our findings should be interpreted alongside several considerations. First, the cross-sectional design limited our ability to quantify intra-individual variability and capture longitudinal trajectories ^99^. Therefore, age- and group-by-age–associated CSF-based alterations should be interpreted cautiously. Longitudinal studies spanning the lifespan are needed to clarify the role of CSF-based mechanisms in neurobiological vulnerability contributing to increased dementia risk in ASD. Second, the sample primarily comprised cognitively unimpaired autistic adults. While this allowed characterization of predisposed CSF-related vulnerability in middle and older adulthood, it limited our ability to disentangle neurodevelopmental from neurodegenerative processes contributing to pathological aging in ASD. Studies including autistic adults across the dementia spectrum will be essential to address this gap. Third, participants were predominantly of average or above-average intelligence; thus, findings may not generalize to autistic adults with ID. Although ID modifies dementia risk in ASD^2^, our results suggest that ASD itself—independent of ID—may be associated with altered CSF-related neurobiology linked to pathological brain aging. Finally, health factors including sleep disturbance, cardiovascular and metabolic disease, polypharmacy, and lifelong medication exposure, may have influenced these findings. Future large-scale studies should systematically evaluate their potential confounding and moderating effects.

## Conclusions

This study represents the first multimodal MRI investigation of CSF-based markers in ageing autistic adults. We identified convergent impairments of CSF–based clearance mechanisms in ASD, including region-specific alterations in white matter perivascular spaces, reduced perivascular fluid diffusivity, and lateral ventricular enlargement. Considered alongside prior findings in autistic children,^54,57^ these results support a lifelong pattern of altered CSF regulation that extends into the pathologically vulnerable periods of middle and older adulthood. Collectively, these findings establish a conceptual foundation for future research aimed at disentangling lifelong neurobiological vulnerabilities that may contribute to increased dementia risk in ASD.

## Supplementary Material

Supplementary Files

This is a list of supplementary files associated with this preprint. Click to download.

• PVS.Supplementary.Tables.02222026.docx

## Figures and Tables

**Table 1:** Demographic characteristics between autistic participants (ASD) and neurotypical controls (NT)

		ASD	NT	*t/χ* ^*2*^	*p*
**Sample size (n)**	**49**	**61**	**–**	**–**	
Age (years)^a^	46 ± 11	48 ± 12	0.65	0.52	
Range	30 – 73	30 – 70	–	–	
Sex (M/F)	28/21	29/32	1.00	0.32	
FSIQ score^a^	107 ± 14	108 ± 12	0.58	0.56	
Range	77 – 143	82 – 126	–	–	
vIQ score^a^	107 ± 14	106 ± 11	-0.27	0.79	
Range	71 – 136	83 – 130	–	–	
pIQ score^a^	105 ± 14	108 ± 15	1.25	0.21	
Range	72 – 142	79 – 142			
eTIV^a^	1545 ± 149	1512 ± 157	-1.15	0.25	
Relative head motion (mm)	0.46 ± 1.08	0.30 ± 0.67	0.09	0.38	
ADOS-2 SA score^b^	9 ± 3	–	–	–	
Range	5 – 18	–	–	–	
ADOS-2 RRB^b^	2 ± 1	–	–	–	
Range	0 – 6	–	–	–	

a Age, FSIQ, pIQ, vIQ and eTIV shown as mean and standard deviation

b ADOS-2 Social Affect (SA) and Restricted and Repetitive Behavior (RRB) are derived from the Module 4 revised algorithm and shown as mean and standard deviation

**Table 2:** Linear regression results of p-perm < 0.05 WM-PVS metrics

Metric	Lobe	Mask	Term	*β*	SE	*t*	*p-perm*
DM	Frontal	L-parsopercularis	Group⋅Age	0.0066	0.0030	2.21	0.029
DM		L-caudalmiddlefrontal	Age	0.0041	0.0016	2.62	0.010
VF		L-caudalmiddlefrontal	Age	0.0001	0.0001	2.48	0.017
VF		L-parsopercularis	Age	0.0002	0.0001	3.01	0.002
DM		L-parstriangularis	Age	0.0041	0.0015	2.74	0.009
VF		L-precentral	Age	0.0001	0.0000	2.30	0.025
DM		L-rostralmiddlefrontal	Age	0.0039	0.0012	3.15	0.003
VF		L-superiorfrontal	Age	0.0001	0.0001	1.96	0.047
DM		R-parsopercularis	Age	0.0045	0.0020	2.22	0.029
VF		R-parsopercularis	Age	0.0001	0.0001	2.45	0.015
CF		R-parstriangularis	Age	0.0000	0.0000	2.64	0.011
DM		R-parstriangularis	Age	0.0054	0.0018	2.98	0.005
**VF**		**R-parstriangularis**	**Age**	**0.0002**	**0.0000**	**3.75**	**0.000**
VF		R-precentral	Age	0.0001	0.0000	2.08	0.039
DM		R-rostralmiddlefrontal	Age	0.0030	0.0010	2.97	0.004
VF		R-superiorfrontal	Age	0.0001	0.0000	2.27	0.026
CF	Parietal	R-inferiorparietal	Group	0.0002	0.0001	1.98	0.048
CF		R-supramarginal	Group	0.0003	0.0001	2.41	0.019
CF		L-inferiorparietal	Group⋅Age	0.0000	0.0000	2.68	0.010
DM		L-inferiorparietal	Group⋅Age	0.0069	0.0032	2.19	0.032
**VF**		**L-inferiorparietal**	**Group⋅Age**	**0.0003**	**0.0001**	**3.14**	**0.002**
DM		L-precuneus	Group⋅Age	0.0061	0.0023	2.68	0.008
VF		L-precuneus	Group⋅Age	0.0001	0.0001	2.07	0.046
CF		L-precuneus	Age	0.0000	0.0000	−2.01	0.045
DM		L-supramarginal	Age	0.0040	0.0018	2.19	0.028
DM		R-inferiorparietal	Age	0.0048	0.0020	2.37	0.023
VF		R-postcentral	Age	0.0001	0.0000	2.28	0.026
DM		R-precuneus	Age	0.0029	0.0015	1.96	0.048
VF		R-supramarginal	Age	0.0001	0.0000	2.46	0.019
CF	Temporal	L-entorhinal	Group	−0.0004	0.0002	−2.13	0.037
DM		L-parahippocampal	Group	−0.4256	0.1644	−2.59	0.011
CF		R-transversetemporal	Group	0.0009	0.0004	2.48	0.014
VF		R-transversetemporal	Group	0.0034	0.0015	2.32	0.021
DM		L-fusiform	Group⋅Age	0.0050	0.0024	2.11	0.038
VF		L-inferiortemporal	Group⋅Age	0.0001	0.0001	2.39	0.021
DM		L-inferiortemporal	Age	0.0027	0.0012	2.27	0.024
CF		L-middletemporal	Age	0.0000	0.0000	2.07	0.041
VF		L-middletemporal	Age	0.0001	0.0000	2.23	0.028
VF		R-inferiortemporal	Age	0.0001	0.0000	2.06	0.040
VF		R-middletemporal	Age	0.0001	0.0000	2.36	0.021
DM	Occipital	L-lingual	Group	−0.3454	0.1623	−2.13	0.036
VF		L-pericalcarine	Group	−0.0016	0.0008	−1.98	0.048
DM		L-lingual	Age	−0.0198	0.0093	−2.13	0.037

Bold indicates significance after Bonferroni correction

DM = Diameter mean; VF = volume fraction; CF = count fraction; L = Left; R = Right

**Table 3:** Linear regression results of DTI-ALPS index

Metric	Term	*β*	SE	*t*	*p-perm*
DTI-ALPS index	**Group**	**−0.0624**	**0.0282**	**−2.21**	**0.029**
	Group⋅Age	0.0025	0.0025	1.01	0.322
	**Age**	**−0.0043**	**0.0016**	**−2.63**	**0.011**

Bold indicates significance after Bonferroni correction

**Table 4:** Linear regression results of lateral ventricles and choroid plexus volumes

Metric	Term	*β*	SE	*t*	*p-perm*
L-Lateral ventricle	**Group**	**0.0018**	**0.0006**	**2.76**	**0.010**
	Group*Age	0.0000	0.0001	0.33	0.751
	**Age**	**0.0001**	**0.0000**	**2.97**	**0.003**
R-Lateral ventricle	**Group**	**0.0018**	**0.0006**	**3.09**	**0.001**
	Group*Age	0.0001	0.0001	1.48	0.142
	**Age**	**0.0001**	**0.0000**	**2.35**	**0.018**
L-Choroid plexus	**Group**	**0.0000**	**0.0000**	**2.38**	**0.021**
	Group*Age	0.0000	0.0000	−0.08	0.939
	**Age**	**0.0000**	**0.0000**	**2.37**	**0.018**
R-Choroid plexus	**Group**	**0.0000**	**0.0000**	**2.73**	**0.008**
	Group*Age	0.0000	0.0000	1.18	0.242
	Age	0.0000	0.0000	0.97	0.341

Bold indicates significance after Bonferroni correction

L = Left; R = Right
